# Supplementary material for: Surface Microbiome Profiling of Dental Elevators Using Third‐Generation Sequencing: Implications for Infection Control in Dental Practice
Source: Microbiologyopen. 2025 Dec 8;14(6):e70178. doi: 10.1002/mbo3.70178 (PMC12685768; doi:10.1002/mbo3.70178)
Supplement: Supplementary file 1 — customer_backup. [file MBO3-14-e70178-s001.zip › customer_backup/customer_backup/taxa_summary/krona/samples/M07.Krona.html]

Javascript must be enabled to view this page.

members
magnitude
magnitudeUnassigned

M07.krona

47756

47754

48

48

48

48

48

1

4

13

2

13

6

4

5

5056

38

38

38

38

38

182

182

3

3

3

19

19

19

160

54

3

17

21

13

4

4

14

14

30

4

26

14

10

4

41

1

14

26

3

3

378

41

41

21

21

1

1

18

18

1

1

50

18

18

18

9

9

9

3

3

3

4

4

4

12

12

12

4

4

4

24

19

19

19

5

5

5

263

7

1

1

6

6

37

37

3

3

31

156

14

2

12

137

4

133

5

2

3

63

1

1

62

62

2132

1470

2

2

2

511

400

178

49

24

126

23

2

2

71

3

1

67

18

13

5

20

20

957

19

19

934

934

4

4

662

414

414

56

346

12

248

5

5

4

4

239

239

2249

204

204

21

21

183

13

9

16

2

143

1

1

1

1

10

10

10

10

10

10

2

2

8

2

5

1

46

46

1

1

7

1

6

5

5

1

1

10

5

1

1

3

9

1

5

3

8

8

5

5

377

78

12

1

3

8

44

5

7

1

3

4

2

8

2

1

3

3

5

16

16

6

6

299

49

49

2

2

27

27

99

99

2

2

120

120

1

1

1

1

77

29

29

3

26

15

12

12

3

3

33

33

8

8

7

10

188

188

188

2

10

12

77

22

65

130

40

2

2

32

32

6

6

4

1

1

3

3

8

8

4

4

4

4

4

8

8

1

7

66

23

23

20

20

23

23

96

96

1

1

85

2

3

6

9

43

22

1

1

2

1

1

1

1

6

6

1097

9

2

2

2

2

4

4

1

1

249

4

4

37

37

4

4

89

89

47

47

68

16

23

27

2

112

106

103

3

6

6

700

6

6

11

11

16

16

1

1

1

1

2

2

8

8

648

3

35

610

1

1

4

4

1

1

1

1

27

27

27

12

1

1

1

11

11

11

77

77

77

77

1

43

32

1

70

70

70

70

70

21

49

156

156

156

156

156

156

746

746

746

724

101

101

623

623

22

22

22

751

751

751

648

648

16

632

103

103

42

61

18

18

18

11

11

11

7

7

7

303

63

63

1

1

1

29

4

4

25

25

2

2

2

7

3

3

4

4

24

22

22

2

2

240

240

129

129

129

1

1

1

7

7

7

3

3

3

100

100

100

145

140

140

140

119

119

14

1

12

1

7

7

5

5

5

5

5

8

8

8

8

3

3

5

5

226

226

226

4

4

4

1

1

1

220

2

2

187

187

31

31

1

1

1

346

150

17

17

17

17

133

37

31

28

3

4

4

2

2

96

6

6

22

22

5

5

63

63

112

60

60

60

12

34

9

5

2

2

2

2

50

50

41

12

2

27

3

3

6

1

5

6

1

1

1

1

5

5

5

5

11

11

11

11

2

1

4

4

67

67

6

6

6

50

50

50

11

11

11

1

1

1

1

1

1

172

45

45

45

1

1

17

17

9

9

18

9

9

127

127

127

13

13

103

39

1

63

4

2

2

7

7

6

6

6

6

1

1

5

5

610

83

83

83

83

83

527

124

112

89

17

63

9

23

3

18

2

12

12

11

1

393

3

3

3

390

390

7

383

10

10

1

1

9

2

7

6

6

6

3

3

3

3

3

1

1

1

52

34

16

16

3

3

13

9

4

18

14

14

14

1

1

1

3

3

3

15

15

15

15

15

3

3

3

2

2

1

1

12623

5343

94

94

54

54

37

37

3

3

76

2

2

2

74

2

2

1

1

1

1

70

6

64

187

187

4

4

2

2

181

181

44

44

1

1

43

1

16

26

4

4

4

4

331

331

15

3

8

4

36

2

3

19

1

2

9

70

3

54

9

4

2

2

20

8

2

10

188

2

1

20

2

1

7

31

16

10

24

3

18

35

6

9

1

2

2391

2391

1

1

7

3

4

2225

2225

43

17

18

8

80

2

7

2

1

2

66

28

11

14

3

7

7

1365

54

34

3

31

4

2

2

16

5

5

6

14

14

14

58

6

1

5

38

5

5

1

27

14

14

5

5

5

4

4

4

2

2

2

57

5

5

52

9

35

1

7

11

11

11

51

42

42

9

9

319

6

3

3

47

47

93

93

16

15

1

1

1

156

16

140

210

39

35

4

3

3

2

2

25

25

71

1

53

5

11

1

70

4

2

7

15

1

10

31

44

6

6

3

3

20

20

10

10

5

5

35

35

2

33

501

151

151

9

1

1

7

1

1

19

6

11

2

8

8

239

11

34

2

76

1

10

96

4

5

74

74

72

4

2

2

2

2

68

14

1

1

6

6

5

5

3

3

11

11

35

34

1

1

1

1

1

13

2

2

2

2

2

2

1

1

1

3

3

3

2

2

2

1

1

1

2

2

2

765

765

9

8

1

1

1

514

73

30

121

290

15

2

13

1

1

203

24

38

141

22

22

7280

11

11

11

11

2345

2

2

2

4

4

4

25

25

25

25

25

25

994

3

3

5

5

839

839

90

32

3

28

27

57

3

11

1

4

1

2

7

15

13

34

6

6

1

1

7

2

3

2

16

16

3

3

1

1

2

2

2

34

1

1

1

1

10

9

1

22

14

8

8

7

7

1

1

686

1

1

2

2

16

16

7

7

2

2

8

3

5

35

2

14

19

32

16

16

58

58

5

5

80

80

440

440

15

15

15

516

20

20

496

496

1592

4

2

2

2

2

475

365

365

31

31

27

27

52

52

59

59

59

2

2

2

1

1

1

1051

1051

1051

35

34

15

11

4

18

18

1

1

1

1

1

21

21

21

2

10

9

38

38

38

38

17

17

17

17

1

1

1

1

22

22

22

21

1

45

2

2

2

43

8

8

1

1

23

8

15

11

2

2

2

2

2

1

3153

1

1

1

18

18

18

43

43

24

17

2

1

1

1

3090

1

1

3069

3069

20

1

19

1

1

1

1

1

1

23

23

23

23

23

5

8

10

181

100

100

1

1

1

99

21

21

9

9

4

4

8

8

3

3

8

1

7

46

44

1

1

81

37

16

9

8

1

1

1

6

2

2

2

1

1

1

6

6

6

14

6

6

8

8

44

44

44

2

17

25

15010

8107

17

17

16

12

3

1

1

1

21

21

10

10

11

11

41

21

21

21

20

20

20

6

6

6

6

6579

6579

5

5

40

40

45

7

38

100

33

67

7

7

9

9

2

2

8

1

7

5

4

1

189

2

4

7

4

2

3

1

17

84

9

2

3

1

6

3

41

4

4

22

22

269

108

1

151

9

53

5

47

1

125

18

3

96

8

1286

17

2

1230

37

95

95

178

178

1

1

27

27

347

347

5

5

54

7

3

25

19

2

2

16

16

2

2

8

8

9

1

8

1

1

22

1

21

19

6

13

3585

9

303

33

142

2909

6

6

158

19

39

39

272

127

40

7

8

25

1

1

42

42

38

29

9

6

6

1

1

1

107

2

2

57

57

4

4

1

1

43

35

8

37

37

37

8

8

7

3

1

2

1

1

1

272

85

85

85

14

14

14

173

173

173

874

436

2

2

5

5

3

1

2

20

2

7

10

1

46

46

23

17

2

4

3

3

3

3

8

8

286

286

37

2

8

27

162

162

162

234

82

54

28

3

3

20

7

4

1

8

78

78

38

3

5

1

13

16

7

7

6

5

1

42

2

1

1

35

6

29

5

5

17

3

3

3

14

14

14

6808

8

8

8

8

749

552

552

18

1

1

15

469

17

1

7

15

8

197

9

9

7

7

4

4

32

25

6

1

74

4

70

15

15

55

55

1

1

2

2

2

2

3079

693

26

26

601

39

486

76

63

15

48

3

3

2386

1026

703

323

26

26

17

17

89

89

778

154

624

65

65

90

90

4

4

267

236

31

11

11

10

10

3

3

47

44

2

2

17

17

25

14

11

3

3

2

1

103

103

87

3

2

5

1

6

11

9

3

36

1

9

1

3

3

13

11

2

2

2

2

2

18

3

3

3

3

3

3

12

12

12

1998

1998

1998

1993

5

791

58

54

48

5

1

4

4

101

99

8

8

8

23

42

8

1

1

2

2

12

12

4

8

617

4

4

3

3

12

3

5

4

1

1

169

93

4

72

18

18

272

190

40

42

8

8

118

1

117

4

3

1

5

5

3

3

3

3

3

7

7

7

7

4

4

3

3

1

1

3

3

3

3

3

91

91

60

3

3

57

38

19

1

1

1

26

3

3

19

15

4

4

4

4

4

4

1

1

1

1

1

48

4

2

2

2

2

2

1

1

1

1

1

1

25

3

3

3

3

9

9

5

5

4

1

3

1

1

1

1

8

8

8

8

4

4

4

4

19

19

19

19

19

108

108

108

108

108

9

99

7

7

7

7

7

5

2

63

49

49

48

48

48

1

1

1

14

14

14

8

4

4

3

3

3

1

2

10894

10891

228

3

3

3

225

1

1

63

5

21

37

27

27

13

9

2

2

9

9

59

14

6

4

7

4

17

3

3

1

14

8

2

4

3

3

1

1

4

4

12

12

8

8

11

11

82

71

41

1

22

18

30

30

11

11

11

10446

3997

152

152

241

241

71

71

3533

3533

89

2

2

87

2

1

3

7

13

7

5

49

18

18

18

42

2

2

40

1

5

34

1

1

1

53

53

53

1788

1788

7

809

144

739

12

3

24

50

761

17

17

16

16

728

448

24

20

224

12

3697

2878

2878

180

180

549

549

12

12

78

56

13

3

6

7

7

5

2

3

2

2

128

25

3

3

20

6

12

2

2

2

28

1

1

15

3

12

6

6

6

6

11

11

6

5

12

4

4

8

8

52

43

1

5

7

20

6

4

6

3

3

1

1

2

1

1

1

1

1

1

1

2

2

2

2

2

75

75

75

9

9

9

13

13

13

53

53

53

1

1

1

1

1

1

2

1

1

1

1

1

1

1

1

1

1

1

1
